# Reconstruction of evolutionary trajectories of chromosomes unraveled independent genomic repatterning between Triticeae and *Brachypodium*

**DOI:** 10.1186/s12864-019-5566-8

**Published:** 2019-03-07

**Authors:** Zhenyi Wang, Jinpeng Wang, Yuxin Pan, Tianyu Lei, Weina Ge, Li Wang, Lan Zhang, Yuxian Li, Kanglu Zhao, Tao Liu, Xiaoming Song, Jiaqi Zhang, Jigao Yu, Jingjing Hu, Xiyin Wang

**Affiliations:** 1 School of Life Sciences, North China University of Science and Technology, Tangshan, 063210 Hebei China; 2 Center for Genomics and Computational Biology, North China University of Science and Technology, Tangshan, 063210 Hebei China; 3 College of Science, North China University of Science and Technology, Tangshan, 063210 Hebei China

**Keywords:** Wheat, Barley, *Brachypodium*, Chromosome, Telomere, Grass

## Abstract

**Background:**

After polyploidization, a genome may experience large-scale genome-repatterning, featuring wide-spread DNA rearrangement and loss, and often chromosome number reduction. Grasses share a common tetraploidization, after which the originally doubled chromosome numbers reduced to different chromosome numbers among them. A telomere-centric reduction model was proposed previously to explain chromosome number reduction. With Brachpodium as an intermediate linking different major lineages of grasses and a model plant of the Pooideae plants, we wonder whether it mediated the evolution from ancestral grass karyotype to Triticeae karyotype.

**Results:**

By inferring the homology among Triticeae, rice, and Brachpodium chromosomes, we reconstructed the evolutionary trajectories of the Triticeae chromosomes. By performing comparative genomics analysis with rice as a reference, we reconstructed the evolutionary trajectories of Pooideae plants, including *Ae. Tauschii* (2n = 14, DD), barley (2n = 14), *Triticum turgidum* (2n = 4x = 28, AABB), and *Brachypodium* (2n = 10). Their extant Pooidea and Brachypodium chromosomes were independently produced after sequential nested chromosome fusions in the last tens of millions of years, respectively, after their split from rice. More frequently than would be expected by chance, in *Brachypodium*, the ‘invading’ and ‘invaded’ chromosomes are homoeologs, originating from duplication of a common ancestral chromosome, that is, with more extensive DNA-level correspondence to one another than random chromosomes, nested chromosome fusion events between homoeologs account for three of seven cases in *Brachypodium* (*P*-value≈0.00078). However, this phenomenon was not observed during the formation of other Pooideae chromosomes.

**Conclusions:**

Notably, we found that the *Brachypodium* chromosomes formed through exclusively distinctive trajectories from those of Pooideae plants, and were well explained by the telomere-centric model. Our work will contribute to understanding the structural and functional innovation of chromosomes in different Pooideae lineages and beyond.

**Electronic supplementary material:**

The online version of this article (10.1186/s12864-019-5566-8) contains supplementary material, which is available to authorized users.

## Background

Whole-genome duplication (WGD) occurs recursively and shapes the plant genomes. Ploidy changes have been quite common during cereal evolution [1]. The origination of cereals were related to a paleopolyploid event ~ 100 million years ago (Mya). Cereals are the major food in temperate regions. Their genomes are characterized by a high content of repetitive elements, such as the Triticeae plants, barley and wheat.

Wheat is now one of the most widely cultivated crops [2], and was domesticated in the Fertile Crescent more than 10,000 years ago [3, 4]. It executes a diploid inheritance but has a genome of an ancestral hexaploid origin, resulting from the union of three diploid grasses [5]. A hybridization of the tetraploid durum wheat (*Triticum turgidum*; AABB; 2n = 4x = 28) with the wild diploid grass (*Aegilops tauschii*; DD; 2n = 2x = 14) resulted in hexaploid wheat (*Triticum aestivum*; AABBDD; 2n = 6x = 42, [6–8]). A 10.1-gigabase assembly of the 14 chromosomes of wild tetraploid wheat was reported in 2017 [3]. The Genome of wild wheat progenitor *Triticum dicoccoides* was sequenced in 2018 [9]. The complex polyploidy nature of wheat large genomes brings difficulty of genetic and functional analyses [10]. By use of wheat ancestors, the approach would provides a viable alternative to overcome the complex polyploidy challenging [11]. The wheat diploid progenitor species *Triticum urartu* (AA) [10, 12], *Aegilops tauschii* (DD) [7, 13, 14], and tetraploid wheat *Triticum turgidum* (AABB) [3, 9] provides convenience for studying the evolution of the wheat genome structure changes. Barley (*Hordeum valgare*) is among the earliest domesticated crops. A high-quality reference genome assembly for barley was presented [15], and the repetitive fraction of the 5100 Mb barley genome was analyzed in 2017 [16]. Actually, both genetic research and crop improvement in barley have benefited from genome sequencing [17].

Owing to its small and conservative genome, rice proved to be a model for other monocotyledonous species. It was sequenced as the second plant genome, and reported to have evolved much slower than other grasses, and preserved the ancestral genome structure after the grass-common whole-genome duplication (cWGD) [18, 19]. *Brachypodium distachyon* (*Brachypodium*) is a member of the Pooideae subfamily, its morphological and genomic features make it a model monocot plant for both comparative and functional genomics for its Pooideae relatives [20–25]. It has a small and compact genome, self-fertility, a life cycle of less than 4 months, and undemanding growth requirements [25, 26]. Besides, it is phylogenetically close to barley and wheat [25]. Due to the availability of its genome sequence [27] and many tools for functional genomics, *Brachypodium* was proposed to be used as a model for genomes of all temperate grasses [28]. The molecular cytogenetic studies advanced greatly with the development of *Brachypodium* bacterial artificial chromosome (BAC) libraries [29]. These resources coupled with the sequenced genome of *Brachypodium* provided insight into grass karyotype evolution [30]. *Brachypodium* shares an extensive synteny among other grasses, so it was a good structural model for the assembly of large genomes [28]. *Brachypodium* is also taken as a good intermediate between wheat and rice [31]. The availability of *Brachypodium* pan-genome sequences revealed genes doubled previous inference in an individual genome [32].

During the evolution of grasses, there has been continually genome repatterning, especially after the whole-genome duplications, often followed by genome instability and fractionation. Eukaryotic chromosomes contain linear structure possessing centromeres and telomeres, which keep the integrity of them and prevent chromosome fusions during nuclear divisions. Centromeric sequences may differ between species, while telomeric sequences are usually highly conserved among plants. Karyotype evolution can be resolved by genome sequencing, comparative genetic mapping, and comparative chromosome painting [33]. The *A. thaliana* karyotype evolution was inferred based on comparative chromosome painting in 2006 [33]. It was proposed that chromosome number reduction is often the result of reciprocal translocations, which combine two chromosomes into a larger one and a smaller one. The smaller chromosome got lost during meiosis [34]. Whole-genome duplication and erroneous DNA double-strand break repair are the main sources of genome structural variation [35].

Paleogenomics is adapted to reconstruct ancestral genomes from the genomes of actual modern species [36]. Modern genomes arose through centromeric fusion of protochromosomes, leading to neochromosomes [37]. The genome of the common ancestor of flowering plants was reconstructed in 2017 [38]. A new theory of telomere-centric genome repatterning explains chromosome number reductions of linear chromosomes [19], emphasizing the removal of telomeres during the process. Accordingly, evolutionary trajectories of genome repatterning and chromosome changes along some major grass lineages were reconstructed during the last ~ 100 millions of years [18].

So far, the formation and evolutionary trajectory of Triticeae chromosomes, shared by wheat, barley, and other close relatives, have not been available. With Brachpodium as an intermediate linking different major lineages of grasses and a model plant of the Pooideae plants, we wonder whether/how it mediated the evolution from ancestral grass chromosomes to Triticeae chromosomes. Here, by inferring the homology within each genome and between them, we reconstructed the evolutionary trajectories of the Triticeae chromosomes, and compared to those of *Brachypodium* chromosomes. This present work will contribute to understanding the structural and functional innovation of chromosomes in different Pooideae lineages.

## Methods

### Plant genome data sets

The genome of the rice (*Oryza sativa*; 2n = 24) and *Brachypodium* (*Brachypodium distachyon*; 2n = 10) were downloaded from the Phytozome version 12 (https://phytozome.jgi.doe.gov/pz/portal.html). The genome of the wild diploid grass *Aegilops tauschii* (2n = 2x = 14; DD) was downloaded from the GenBank as v4.0 under BioProject PRJNA341983. The genome of the Barley (*Hordeum vulgare*; 2n = 14) was downloaded from the IPK Barley Blast Server (http://webblast.ipk-gatersleben.de/barley_ibsc/). The genome of the tetraploid wheat *Triticum turgidum* (2n = 4x = 28; AABB) was downloaded from the WEWseq (http://wewseq.wixsite.com/consortium).

### Inferring collinear homologs

Grasses share extensive gene collinearity, that is, thouands of genes share the same chromosomal order in the different plants, indicating descent from a common ancestral chromosomal region. To reveal gene colinearity, each genome was compared against other genomes using BLASTP, and also compared against itself. The best five hits meeting an E-value threshold 1 × 10^− 5^ were retrieved. The syntenic regions were grouped to form multiple alignments using MCscan, the homologous pairs were used as the input for MCscan [39]. The default scoring scheme is min (log10 E, 50) match score for one gene pair and 1 gap penalty for each 10 kb distance between any two consecutive gene pairs. The resulting syntenic chains were evaluated using a procedure adopted by ColinearScan [40], and E-value threshold was set to be 1 × 10^− 10^. We enriched the collinear gene data set by inferring more small homologous blocks by running ColinearScan to detect pairwise chromosome homology. In collinearity methods, maximum gap length (mg) is the most important parameter which determines the length, quality and extensiveness of the predicated collinearity. The mg was set to be 40 intervening genes between neighboring genes in collinearity on both chromosomes. Gene clusters that contain 30 or more genes in a chromosome were removed from the present analysis, in that they may algorithcally happer the inference of gene colinearity, especially when they clustered up in a neighboring region [41].

### Dot-plot generation

We used BLASTN to search for CDS anchors (E-value < 1 × 10^− 5^) between every possible pair of chromosomes in multiple genomes. The best, second best, and other matches with E-value >1e-5 were displayed in different colors, to help distinguish orthology from paralogy, or layers of paralogy as a result of recursive WGD events. Gene families with > 30 members were removed from the analysis, for gene redundancy may lead to an aberrantly fast evolutionary rate and affect the accuracy of analytical results. Dot-plots were produced using Perl scripts [19].

### Flash cartoon production

We used Adobe Flash language to produce flash multimedia cartoons. The seven ancestral chromosomes in seven different colors were related to extant and intermediate chromosomes in different grasses. These color schemes was integrated previous color schemes for grasses [19]. These color schemes were also used in dot-plots.

### Statistical significance of homoeologous chromosome fusion

We estimated the occurrence probability of nested chromosomal fusions (NCFs) between homoeologous chromosomes with combinatorial statistics. For instance, rice (2n = 24) merged from 14 ancestral chromosomes, or seven ancestral homoeologous chromosome pairs. If merged chromosomes are viewed still as independent chromosomal segments, the probability of this event can be estimated. For example, the occurrence probability of one out of two NCFs between homoeologous chromosomes can be estimated with combinatory formula (7, 1)/(14, 2), where (n, m) is n!/[m!(n – m)!)].

## Results

### Inference of Triticeae karyotype evolution

Parsimony-based phylogenomic analysis can help find and relatively date genomic changes, therefore contribute to clarify karyotype evolution. For example, comparing two grass genomes sharing the 100-mya tetraploidy [18], a single chromosomal inversion in their common ancestor would result in incongruity between paralogous chromosomes in both grasses, but no incongruity between the corresponding orthologous chromosomes, whereas an inversion in a chromosome of one grass genome would lead to incongruity with its orthologous chromosomes in the other grass, and at the mean time incongruity with the outparalog chromosomes. Similarly, the above analysis can infer the occurrence of chromosome fission, fusion, and number reduction.

Here, to understand the evolutionary trajectories of Pooideae chromosomes, we analyzed the syntenic conservation and chromosome rearrangements between the genomes of *Ae. tauschii*, barley, *Triticum turgidum,* and two sequenced grass relatives, rice and *Brachypodium* for comparison. By searching homologous genes within a genome or between different genomes, we drew homologous gene dotplots, which showed orthologous correspondence between these genomes and paralogous correspondence in each genome.

**Fig. 1:**
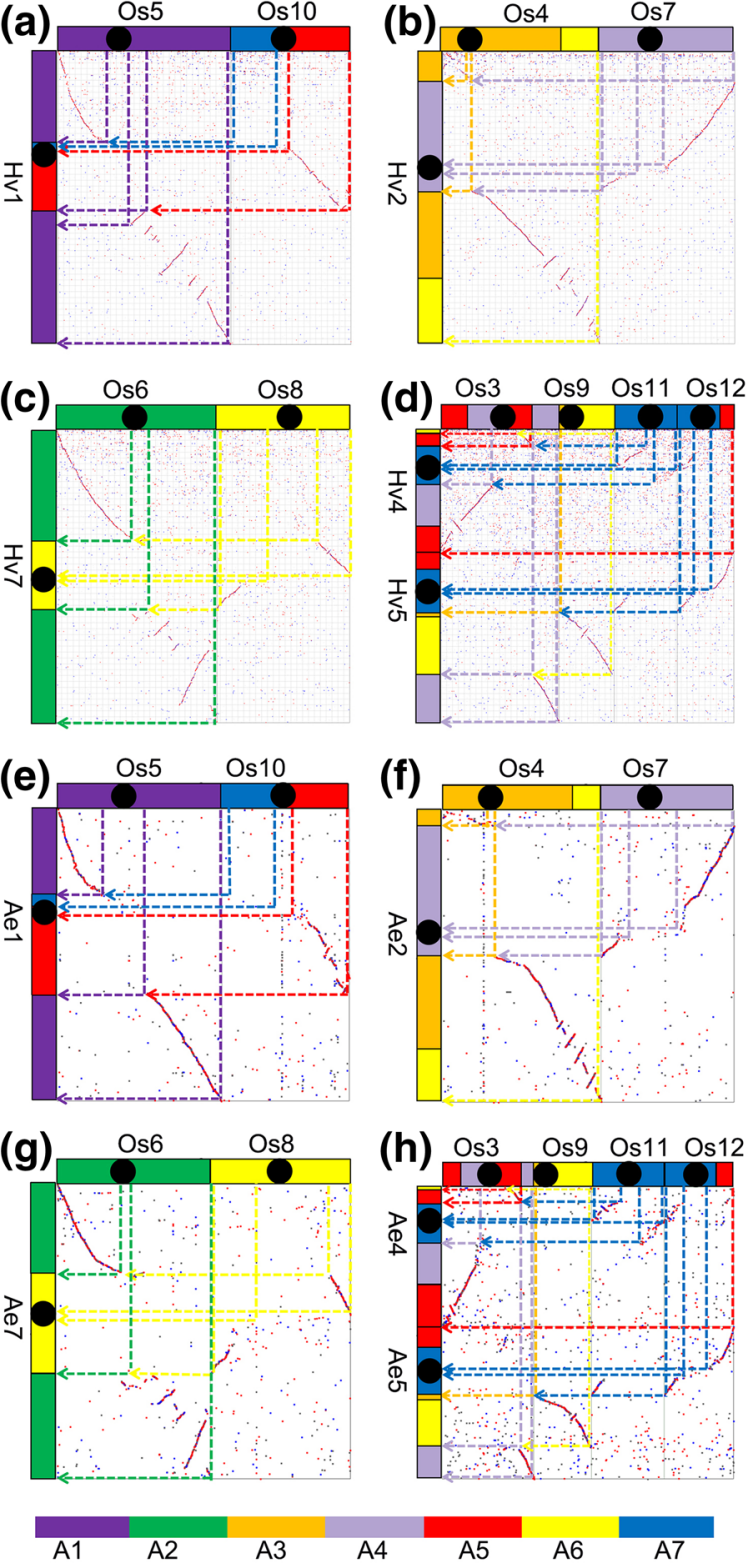
Chromosome fusions during the evolution of *Hordeum vulgare* and *Aegilops tauschii*. Chromosomes, shown as rectangular blocks, are arranged horizontally and vertically to the dot-plot. The color scheme (A1-A7, the seven ancestral chromosomes was used seven different colors as reference were related to chromosomes in different grasses) for the chromosomes of grasses follows that of a previous study [19]. Homologous blocks can be classified as primary, resulting from chromosomal orthology, and secondary, resulting from paralogy from ancestral polyploidy. Hv, *Hordeum vulgare*; Ae, *Aegilops tauschii*; Os, *Oryza sativa*. **a** Formation of chromosome Hv1; **b** formation of chromosome Hv2; **c** formation of chromosome Hv7; **d** formation of chromosome Hv4 and Hv5; **e** formation of chromosome Ae1; **f** formation of chromosome Ae2; **g** formation of chromosome Ae7; **h** formation of chromosome Ae4 and Ae5

**Fig. 2:**
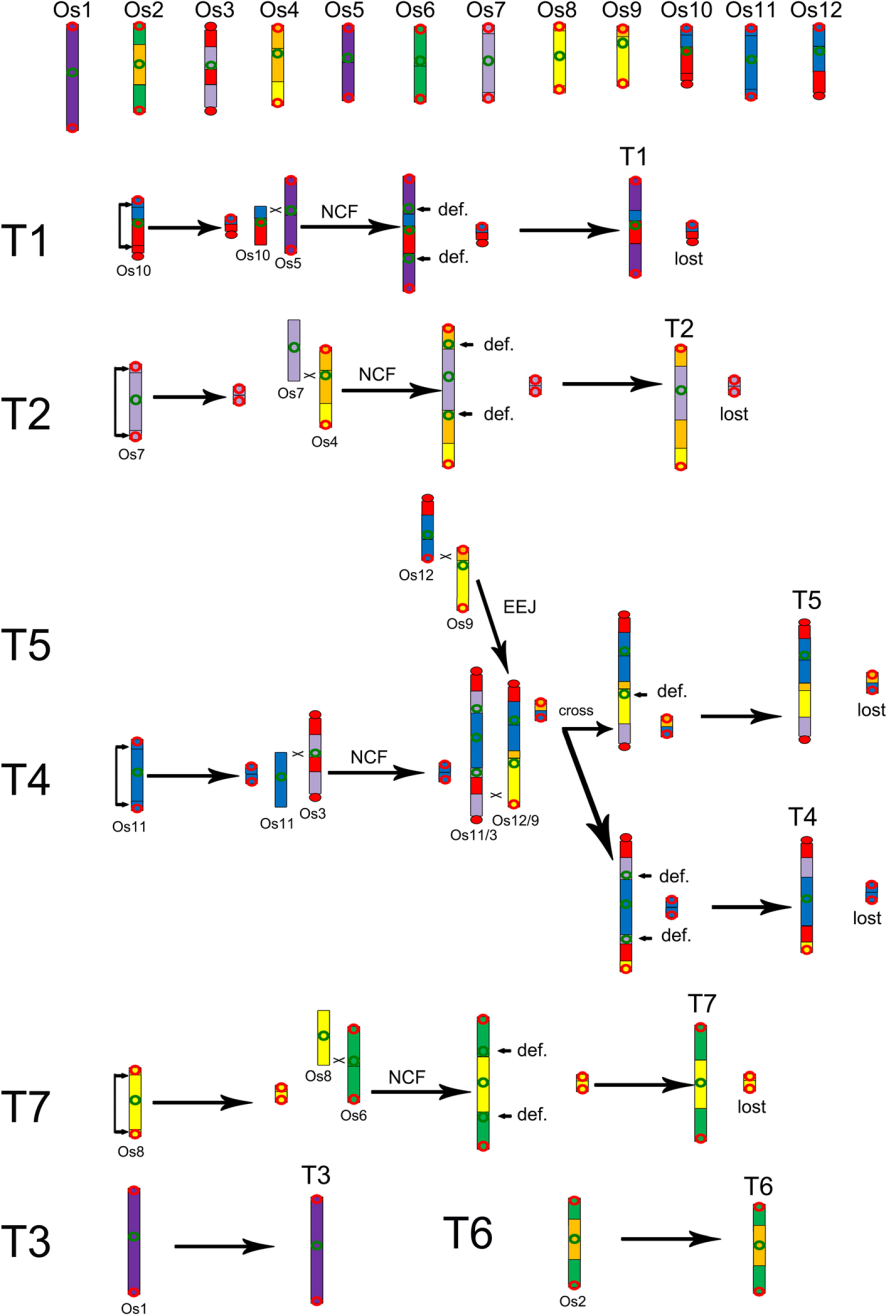
The evolution process of 7 ancestral Triticeae (T) chromosomes

As to homologous gene dotplots between wheat and its Pooideae relatives, we found their 7 chromosomes had nearly perfect orthologous correspondence, showing that they inherited their ancestral karyotype and chromosomes without much changes in chromosome constitution. A homologous dotplot between rice and the Pooideae grasses showed the evolutionary changes that led to chromosome number reduction from 12 in an ancestral haploid grass genome, as previously studied [19]. The 12 ancestral chromosomes were just well represented by extant rice chromosomes with 1–1 correspondence. Therefore, for simplicity, we used rice chromosomes Os1–12 to represent ancestral chromosomes A1–12. Correspondence between orthologous chromosomes or chromosomal segments indicated that Triticeae chromosome 1 (T1) formed by a nested fusion of ancestral chromosome Os10 into chromosome Os5 (Figs. 1a, e and 2). The nested fusion process can occur as follows: Os10 crossed-over to form a major chromosome and a satellite chromosome, then the major chromosome insert the centromeric regions of Os5, the satellite chromosome may be lost. Spatial proximity would then favor ligation, resulting in NCFs.

Likewise, Ae2 (Hv2) formed by a fusion of Os4 and Os7 (Figs. 1b, f and 2), Ae7 (Hv7) formed by a fusion of Os6 and Os8 (Figs. 1c, g and 2). Ae3 (Hv3) and Ae6 (Hv6) were simple, they respectively corresponding to Os1 and Os2. The most complex evolutionary process was Ae4 (Hv4) and Ae5 (Hv5). A fusion of Os11 and Os3 formed an intermediate Os11/3 by nested chromosome fusion (NCF), another intermediate Os12/9 formed by Os12 and Os9 with end–end joining (EEJ), that produced a satellite chromosome, reciprocal translocation of arms between the two intermediates produced extant chromosomes Ae4 (Hv4) and Ae5(Hv5) (Figs. 1d, h and 2). The chromosome evolutionary process of *Triticum turgidum* was the same as *Ae. Tauschii* and barley from the dot-plot between *Triticum turgidum* and rice (Additional file 1: Figure S1). The evolution process of Triticeae is represented in the form of graphs and a video (Fig. 2; Additional file 2: Video S1).


**Additional file 2: Video S1.** Dynamic changes during grass genome repatterning. (MP4 4887 kb)


During the formation of Triticeae chromosomes, four intra-chromosome telomere-proximal crossing occurred to produce four free-end intermediate chromosomes (Fig. 2), which fused into the peri-centromeric regions of other chromosomes, and four satellite chromosomes; and one inter-chromosome telomere-proximal crossing occurred to produce an end-end merging chromosome and a satellite chromosome. The total five satellite chromosomes were all lost, and reduced the chromosome number from 12 to 7 in extant Triticeae genomes. Besides, an inter-chromosome in-arm crossing-over occurred, to exchange DNA between two chromosomes.

### A comparison of karyotype evolution in Pooideae

**Fig. 3:**
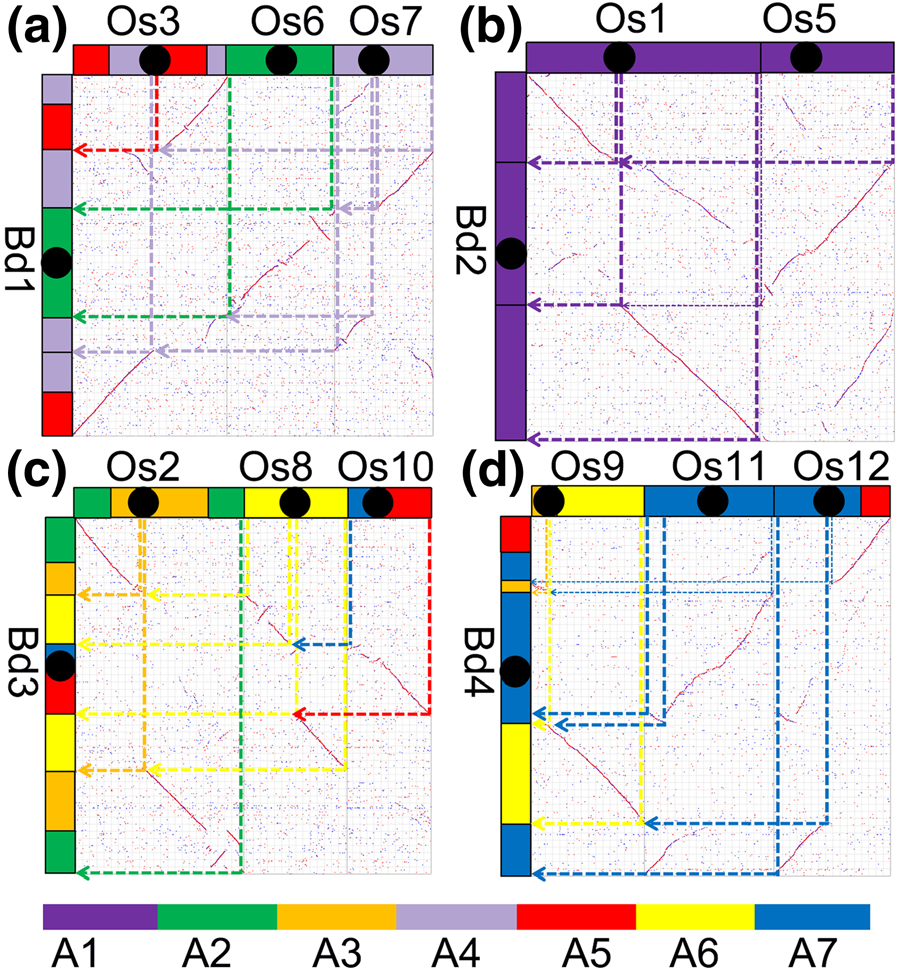
Chromosome fusions during the evolution of *Brachypodium*. Chromosomes, shown as rectangular blocks, are arranged horizontally and vertically to the dot-plot. The color scheme (A1-A7, the seven ancestral chromosomes was used seven different colors as reference were related to chromosomes in different grasses) for the chromosomes of grasses follows that of a previous study [19]. Homologous blocks can be classified as primary, resulting from chromosomal orthology, and secondary, resulting from paralogy from ancestral polyploidy. Bd, *Brachypodium distachyon*; Os, *Oryza sativa*. **a** Formation of chromosome Bd1; **b** formation of chromosome Bd2; **c** formation of chromosome Bd3; **d** formation of chromosome Bd4

**Fig. 4:**
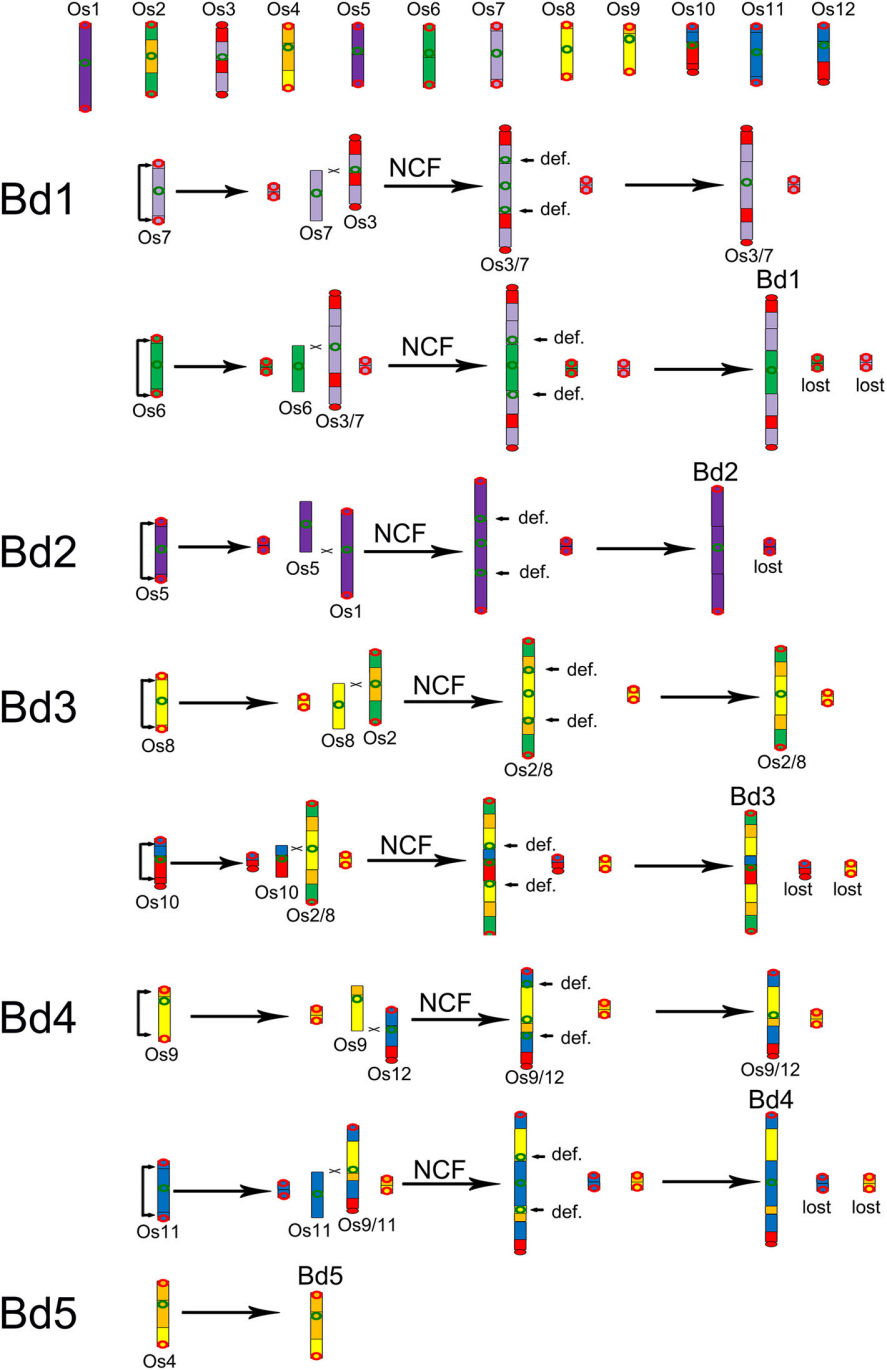
The evolution process of *Brachypodium distachyon* (Bd) chromosomes

As reported previously, the extant *Brachypodium* chromosomes (Bd1–5) formed exclusively by recursive occurrence of NCFs (*Brachypodium* genome sequencing project), and resulted in formations of 7 satellite chromosomes [19]. Here we showed the evolutionary process by following the telomere-centric model. Bd1 formed by two fusions, a fusion of Os3 and Os7 formed an intermediate Os3/7 by NCF, and then, intra-chromosome crossed-over at the proximal regions of two Os6 telomeres produced a major chromosome and a satellite chromosome. One of the sticky ends of Os6 intermediate attached to the peri-centromeric regions of Os3/7 intermediate through NCF to form Bd1, eventually (Figs. 3a and 4).

Bd2 formed by a NCF of Os5 into Os1. Bd3 formed also by two NCFs, with Os8 and Os10 nested into Os2 sequentially in time, or in reversed order (Figs. 3c and 4). Bd4 formed also by two NCFs, with Os11 and Os12 nested into Os9 (Figs. 3d and 4). Bd5 preserved the structure of Os4 (Fig. 4).

During formation of five extant *Brachypodium* chromosomes, seven nested chromosome fusions occurred, to produce seven satellite chromosomes. The loss of these satellite chromosomes resulted in the chromosome number reduction from 12 to 5.

### Distinct evolutionary pathways taken by chromosomes *Brachypodium* and its Pooideae relatives

Notably, the above inference of the evolutionary trajectories of Pooideae chromosomes showed that the karyotypes of *Brachypodium* and its Pooideae relatives under consideration formed totally independently (Figs. 2 and 4). This means that not a single event, e.g., crossing-over or fusion, to form intermediate or extant chromosomes, was shared by two lineages.

Besides, in *Brachypodium*, more frequently than would be expected by chance, the ‘invading’ and ‘invaded’ chromosomes are homoeologs, originating from duplication of a common ancestral chromosome, that is, with more extensive DNA-level correspondence to one another than random chromosomes. In *Brachypodium*, three out of seven NCF events occurred between homoeologous chromosomes, and the corresponding probability can be estimated by (7, 1, 6, 1, 5, 1)/[(14,2, 12, 2, 10, 2)], where (n, m) is n!/[m!(n–m)!], or *P*-value≈0.00078. However, this phenomenon was not observed during the formation of other Pooideae chromosomes. Just none homoeologous fusion occurred to produce Pooideae chromosomes. These suggest that chromosomes in two lineages evolved in exclusively different trajectories.

### Ancestral genome reconstruction

**Fig. 5:**
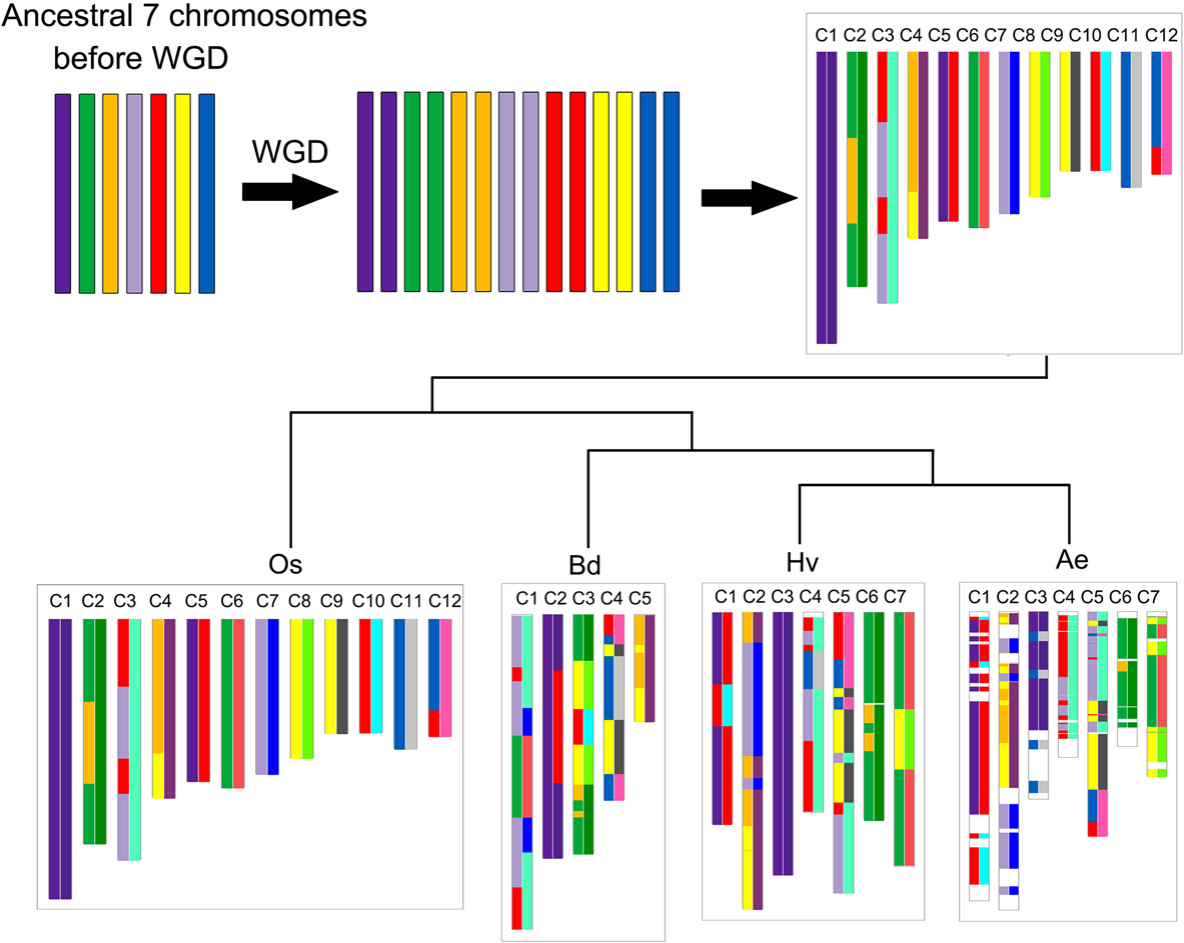
Schematic representation of homologous regions in grass genomes. The seven ancestral chromosomes and twelve rice chromosomes was used as reference were related to chromosomes in different grasses, including *Oryza sativa* (Os), *Brachypodium distachyon* (Bd), *Hordeum vulgare* (Hv), *Ae. Tauschii* (Ae). Each colored block in extant genome corresponds to a homologous region in a referenc genome. An extant chromosome is displayed in two color schemes, with the blocks in left and right, respectively corresponding to the ancestral or the rice chromosomes

By checking gene collinearity, we revealed homologous genes within Triticeae genome, and between it and other grass relatives. Most of the collinear genes with Triticeae were produced by the grass cWGD [42, 43]. Here we used two methods to show the collinearity information. On the one hand, the putative 7 ancient chromosomes was inferred with collinear genes in paralogous regions in a genome, as shown previously [19], and by using these preserved genes to relate to extant chromosomal regions (Fig. 5). On the other hand, rice genes on its 12 chromosomes were related to other genomes to show the collinearity/orthology between them (Fig. 5). These two representation schemes helped find homologous regions between genomes and display evolutionary repatterning results.

## Discussion

### A different evolutionary history

Integrated synteny and phylogenomic analyses of grass genomes had revealed ancient polyploidy events and lineage-specific WGD events [44]. WGD events have been of central importance in angiosperm macroevolution and have provided raw material for natural selection [45, 46]. Poaceae was profoundly influenced by a WGD event that occurred ~ 100 Mya [18]. Following the WGD, genomic instability increased by extensive chromosomal rearrangements and numerous gene losses [42, 43, 47, 48]. These changes eventually led to the formation of a new diploid karyotype [33, 36, 49]. Factors including gene loss, chromosomal rearrangement events and repeat-rich sequence accumulation may have contributed to the evolutionary history, which have to be left for future exploration.

The evolution of chromosome number in organisms is caused by the rearrangement of centromeres and telomeres [50]. The mechanism of chromosome number changes have been studied in certain eukaryotes, such as the fusion of two chromosomes and the insertions of whole chromosomes into other centromeres [51–56]. As to chromosome number reduction, we previously proposed a telomere-centric model to explain likely mechanisms, emphasizing the role of telomeres during the process [19]. Telomeres were inferred to be removed from the same chromosome by forming an intermediate free-end chromosome, which would eventually insert into another chromosome, or from two different chromosomes, the major structure of which would fuse to produce a larger chromosome. During the process, a satellite chromosome, formed by the two removed telomeres and some intervening DNA would be produced. If the satellite chromosome was lost or not counted, that would explain chromosome number reduction.

Chromosomes evolved along exclusively diffrerent trajectories in two studied lineages: Pooideae and Brachypodium. Actually, in that Brachypodium was taken as a model of Triteceae plants, we would anticipated that chromosomes in two lineages may share much of their evolution. Interestingly, we found that Triticeae chromosomes were produced by sequential occurrence of 4 NCFs and 1 chromosome end-end merge, and likely produced 5 satellite chromosomes, while Brachpodium chromosomes were produced 7 NCFs and 7 likely satellite chromosomes. The lost of those satellite chromosomes resulted in chromosome number reduction. Notably, the Pooideae and Brachypodium lineages evolved their extant chromosomes through exclusively different trajectories, that is, not a single event, e.g., crossing-over or fusion, to form intermediate or extant chromosomes, was shared by two lineages. More requently than would be expected by chance, in *Brachypodium*, the ‘invading’ and ‘invaded’ chromosomes are homoeologs, originating from duplication of a common ancestral chromosome, that is, with more extensive DNA-level correspondence to one another than random chromosomes, NCF events between homoeologs account for three of seven cases in *Brachypodium* (*P*-value≈0.00078). However, this phenomenon was not observed during the formation of other Pooideae chromosomes. The situations were completely different along two lineages.

## Conclusions

With Brachpodium as an intermediate linking different major lineages of grasses and a model plant of the Pooideae plants, we wonder whether it mediated the evolution from ancestral grass karyotype to Triticeae karyotype. Notably, we found that the *Brachypodium* chromosomes formed through exclusively distinctive trajectories from those of Pooideae plants, and were well explained by the telomere-centric model. Our work will contribute to understanding the structural and functional innovation of chromosomes in different Pooideae lineages and beyond.

## Additional file

Additional file 1: **Figure S1.** Dot-plot between *Triticum turgidum* and *Oryza sativa*. *Triticum turgidum* and *Oryza sativa* chromosomes are, respectively, aligned horizontally and vertically. Red dots show homologous *Triticum turgidum* genes best matching *Oryza sativa* genes, and blue dots show other matches. WEW, *Triticum turgidum* (2n = 4x = 28; AABB)*.* Os, *Oryza sativa*. WEW (1, 3, 5, 7, 9, 11, 13) are the A genome. WEW (2, 4, 6, 8, 10, 12, 14) are the B genome. (JPG 1989 kb)

## Abbreviations

BAC: Bacterial artificial chromosome
cWGD: Common whole-genome duplication
EEJ: End–end joining
Mya: Million years ago
NCFs: Nested chromosomal fusions
WGD: Whole-genome duplication

## References

[1] Wang H, Yu L, Lai F, Liu L, Wang J. Molecular evidence for asymmetric evolution of sister duplicated blocks after cereal polyploidy. Plant Mol Biol. 2005;59(1):63-74. 10.1007/s11103-005-4414-1 16217602

[2] Brenchley R, Spannagl M, Pfeifer M, Barker GLA, D'Amore R, Allen AM, McKenzie N, Kramer M, Kerhornou A, Bolser D (2012). Analysis of the breadwheat genome using whole-genome shotgun sequencing. Nature.

[3] Avni R, Nave M, Barad O, Baruch K, Twardziok SO, Gundlach H, Hale I, Mascher M, Spannagl M, Wiebe K (2017). Wild emmer genome architecture and diversity elucidate wheat evolution and domestication. Science (New York, NY).

[4] Lev-Yadun S, Gopher A, Abbo S (2000). Archaeology. The cradle of agriculture. Science (New York, NY).

[5] Chalupska D, Lee HY, Faris JD, Evrard A, Chalhoub B, Haselkorn R, Gornicki P (2008). Acc homoeoloci and the evolution of wheat genomes. Proc Natl Acad Sci U S A.

[6] Dubcovsky J, Dvorak J (2007). Genome plasticity a key factor in the success of polyploid wheat under domestication. Science (New York, NY).

[7] Luo MC, Gu YQ, Puiu D, Wang H, Twardziok SO, Deal KR, Huo N, Zhu T, Wang L, Wang Y (2017). Genome sequence of the progenitor of the wheat D genome Aegilops tauschii. Nature.

[8] Salamini F, Ozkan H, Brandolini A, Schafer-Pregl R, Martin W (2002). Genetics and geography of wild cereal domestication in the near east. Nat Rev Genet.

[9] Akpinar BA, Biyiklioglu S, Alptekin B, Havrankova M, Vrana J, Dolezel J, Distelfeld A, Hernandez P, Budak H, IWGSC (2018). Chromosome-based survey sequencing reveals the genome organization of wild wheat progenitor Triticum dicoccoides. Plant Biotechnol J.

[10] Ling HQ, Zhao S, Liu D, Wang J, Sun H, Zhang C, Fan H, Li D, Dong L, Tao Y (2013). Draft genome of the wheat A-genome progenitor Triticum urartu. Nature.

[11] Faricelli ME, Valarik M, Dubcovsky J (2010). Control of flowering time and spike development in cereals: the earliness per se Eps-1 region in wheat, rice, and *Brachypodium*. Funct Integr Genomics.

[12] Ling HQ, Ma B, Shi X, Liu H, Dong L, Sun H, Cao Y, Gao Q, Zheng S, Li Y (2018). Genome sequence of the progenitor of wheat a subgenome Triticum urartu. Nature.

[13] Jia JZ, Zhao SC, Kong XY, Li YR, Zhao GY, He WM, Appels R, Pfeifer M, Tao Y, Zhang XY (2013). Aegilops tauschii draft genome sequence reveals a gene repertoire for wheat adaptation. Nature.

[14] Zhao G, Zou C, Li K, Wang K, Li T, Gao L, Zhang X, Wang H, Yang Z, Liu X (2017). The Aegilops tauschii genome reveals multiple impacts of transposons. Nat Plants.

[15] Mascher M, Gundlach H, Himmelbach A, Beier S, Twardziok SO, Wicker T, Radchuk V, Dockter C, Hedley PE, Russell J (2017). A chromosome conformation capture ordered sequence of the barley genome. Nature.

[16] Wicker T, Schulman AH, Tanskanen J, Spannagl M, Twardziok S, Mascher M, Springer NM, Li Q, Waugh R, Li C (2017). The repetitive landscape of the 5100 Mbp barley genome. Mob DNA.

[17] Mayer KF, Waugh R, Brown JW, Schulman A, Langridge P, Platzer M, Fincher GB, Muehlbauer GJ, Sato K, Close TJ (2012). A physical, genetic and functional sequence assembly of the barley genome. Nature.

[18] Wang X, Wang J, Jin D, Guo H, Lee TH, Liu T, Paterson AH (2015). Genome alignment spanning major Poaceae lineages reveals heterogeneous evolutionary rates and alters inferred dates for key evolutionary events. Mol Plant.

[19] Wang X, Jin D, Wang Z, Guo H, Zhang L, Wang L, Li J, Paterson AH (2015). Telomere-centric genome repatterning determines recurring chromosome number reductions during the evolution of eukaryotes. New Phytol.

[20] Draper J, Mur LA, Jenkins G, Ghosh-Biswas GC, Bablak P, Hasterok R, Routledge AP (2001). *Brachypodium distachyon*. A new model system for functional genomics in grasses. Plant Physiol.

[21] Foote TN, Griffiths S, Allouis S, Moore G (2004). Construction and analysis of a BAC library in the grass *Brachypodium sylvaticum*: its use as a tool to bridge the gap between rice and wheat in elucidating gene content. Funct Integr Genomics.

[22] Huo NX, Vogel JP, Lazo GR, You FM, Ma YQ, McMahon S, Dvorak J, Anderson OD, Luo MC, Gu YQ (2009). Structural characterization of *Brachypodium* genome and its syntenic relationship with rice and wheat. Plant Mol Biol.

[23] Higgins JA, Bailey PC, Laurie DA. Comparative genomics of flowering time pathways using Brachypodium distachyon as a model for the temperate grasses. Plos One 2010;5(4):e10065. 10.1371/journal.pone.0010065 PMC2856676 20419097

[24] Kumar S, Mohan A, Balyan HS, Gupta PK (2009). Orthology between genomes of *Brachypodium*, wheat and rice. BMC Res Notes.

[25] Ozdemir BS, Hernandez P, Filiz E, Budak H (2008). *Brachypodium* genomics. Intern J Plant Genomics.

[26] Huo NX, Lazo GR, Vogel JP, You FM, Ma YQ, Hayde DM, Coleman-Derr D, Hill TA, Dvorak J, Anderson OD (2008). The nuclear genome of *Brachypodium distachyon*: analysis of BAC end sequences. Funct Integr Genomic.

[27] International Brachypodium Initiative (2010). Genome sequencing and analysis of the model grass *Brachypodium distachyon*. Nature.

[28] Budak H, Hernandez P, Schulman AH: Analysis and Exploitation of Cereal Genomes with the Aid of Brachypodium. In: Tuberosa R, Graner A, Frison E, editors. Genomics of Plant Genetic Resources. Springer, Dordrecht; 2014. p. 585–613.

[29] Kerrie F, Donnison IS (2007). Construction and screening of BAC libraries made from *Brachypodium* genomic DNA. Nat Protoc.

[30] Idziakhelmcke D, Betekhtin A (2018). Methods for cytogenetic chromosome barcoding and chromosome painting in *Brachypodium distachyon* and its relative species. Methods Mol Biol.

[31] Kellogg EA (2015). *Brachypodium distachyon* as a genetic model system. Annu Rev Genet.

[32] Gordon SP, Contreras-Moreira B, Woods DP, Des Marais DL, Burgess D, Shu S, Stritt C, Roulin AC, Schackwitz W, Tyler L (2017). Extensive gene content variation in the *Brachypodium distachyon* pan-genome correlates with population structure. Nat Commun.

[33] Lysak MA, Berr A, Pecinka A, Schmidt R, McBreen K, Schubert I (2006). Mechanisms of chromosome number reduction in Arabidopsis thaliana and related Brassicaceae species. Proc Natl Acad Sci U S A.

[34] Schubert I, Lysak MA (2011). Interpretation of karyotype evolution should consider chromosome structural constraints. Trends Genet.

[35] Schubert I, Vu GTH (2016). Genome stability and evolution: attempting a holistic view. Trends Plant Sci.

[36] Salse J, Abrouk M, Bolot S, Guilhot N, Courcelle E, Faraut T, Waugh R, Close TJ, Messing J, Feuillet C (2009). Reconstruction of monocotelydoneous proto-chromosomes reveals faster evolution in plants than in animals. Proc Natl Acad Sci U S A.

[37] Murat F, Zhang R, Guizard S, Flores R, Armero A, Pont C, Steinbach D, Quesneville H, Cooke R, Salse J (2014). Shared subgenome dominance following polyploidization explains grass genome evolutionary plasticity from a seven protochromosome ancestor with 16K protogenes. Genome Biol Evol.

[38] Murat F, Armero A, Pont C, Klopp C, Salse J (2017). Reconstructing the genome of the most recent common ancestor of flowering plants. Nat Genet.

[39] Tang H, Wang X, Bowers JE, Ming R, Alam M, Paterson AH (2008). Unraveling ancient hexaploidy through multiply-aligned angiosperm gene maps. Genome Res.

[40] Wang X, Shi X, Li Z, Zhu Q, Kong L, Tang W, Ge S, Luo J (2006). Statistical inference of chromosomal homology based on gene colinearity and applications to Arabidopsis and rice. BMC Bioinformatics.

[41] Michelmore RW, Meyers BC (1998). Clusters of resistance genes in plants evolve by divergent selection and a birth-and-death process. Genome Res.

[42] Paterson AH, Bowers JE, Chapman BA (2004). Ancient polyploidization predating divergence of the cereals, and its consequences for comparative genomics. Proc Natl Acad Sci U S A.

[43] Wang X, Shi X, Hao B, Ge S, Luo J (2005). Duplication and DNA segmental loss in the rice genome: implications for diploidization. New Phytol.

[44] Ming R, VanBuren R, Wai CM, Tang H, Schatz MC, Bowers JE, Lyons E, Wang ML, Chen J, Biggers E (2015). The pineapple genome and the evolution of CAM photosynthesis. Nat Genet.

[45] Tang H, Bowers JE, Wang X, Ming R, Alam M, Paterson AH (2008). Synteny and collinearity in plant genomes. Science (New York, NY).

[46] Bowers JE, Chapman BA, Rong JK, Paterson AH (2003). Unravelling angiosperm genome evolution by phylogenetic analysis of chromosomal duplication events. Nature.

[47] Sankoff D, Zheng C, Zhu Q (2010). The collapse of gene complement following whole genome duplication. BMC Genomics.

[48] Thomas BC, Pedersen B, Freeling M (2006). Following tetraploidy in an Arabidopsis ancestor, genes were removed preferentially from one homeolog leaving clusters enriched in dose-sensitive genes. Genome Res.

[49] Bowers JE, Arias MA, Asher R, Avise JA, Ball RT, Brewer GA, Buss RW, Chen AH, Edwards TM, Estill JC (2005). Comparative physical mapping links conservation of microsynteny to chromosome structure and recombination in grasses. P Natl Acad Sci USA.

[50] Gordon JL, Byrne KP, Wolfe KH (2011). Mechanisms of chromosome number evolution in yeast. PLoS Genet.

[51] Gordon JL, Byrne KP, Wolfe KH (2009). Additions, losses, and rearrangements on the evolutionary route from a reconstructed ancestor to the modern Saccharomyces cerevisiae genome. PLoS Genet.

[52] Guerra CE, Kaback DB (1999). The role of centromere alignment in meiosis I segregation of homologous chromosomes in Saccharomyces cerevisiae. Genetics.

[53] Hillier LW, Graves TA, Fulton RS, Fulton LA, Pepin KH, Minx P, Wagner-McPherson C, Layman D, Wylie K, Sekhon M (2005). Generation and annotation of the DNA sequences of human chromosomes 2 and 4. Nature.

[54] Ijdo JW, Baldini A, Ward DC, Reeders ST, Wells RA (1991). Origin of human Chromosome-2 - an ancestral telomere telomere fusion. P Natl Acad Sci USA.

[55] Malik HS, Henikoff S (2009). Major evolutionary transitions in centromere complexity. Cell.

[56] Murat F, Xu JH, Tannier E, Abrouk M, Guilhot N, Pont C, Messing J, Salse J (2010). Ancestral grass karyotype reconstruction unravels new mechanisms of genome shuffling as a source of plant evolution. Genome Res.

