# Supplementary figures and images for: Reconstruction of evolutionary trajectories of chromosomes unraveled independent genomic repatterning between Triticeae and Brachypodium
Source: BMC Genomics. 2019 Mar 7;20:180. doi: 10.1186/s12864-019-5566-8 (PMC6407190; doi:10.1186/s12864-019-5566-8)

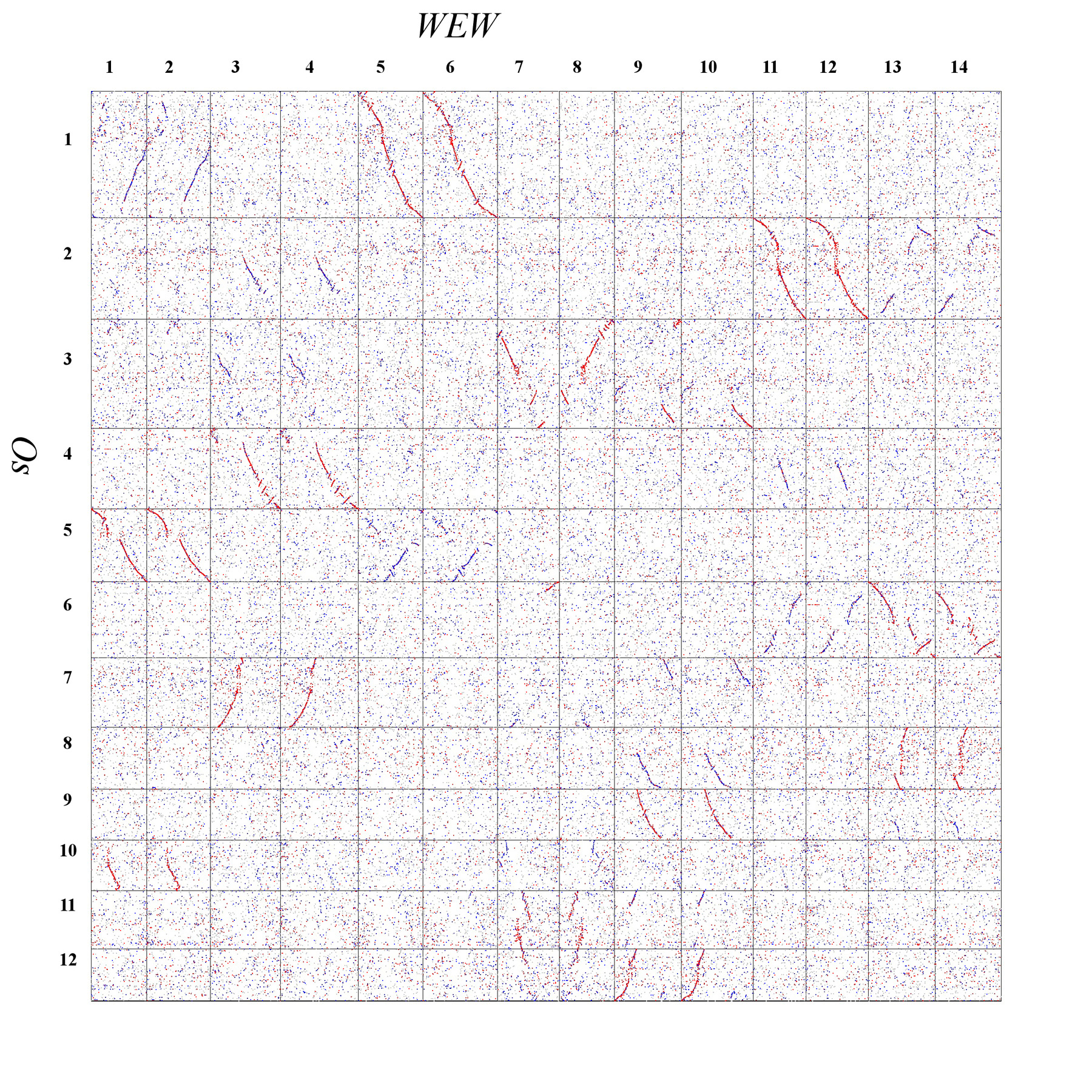

Supplement: Supplementary file 2 — Figure S1. Dot-plot between Triticum turgidum and Oryza sativa. Triticum turgidum and Oryza sativa chromosomes are, respectively, aligned horizontally and vertically. Red dots show homologous Triticum turgidum genes best matching Oryza sativa genes, and blue dots show other matches. WEW, Triticum turgidum (2n = 4x = 28; AABB). Os, Oryza sativa. WEW (1, 3, 5, 7, 9, 11, 13) are the A genome. WEW (2, 4, 6, 8, 10, 12, 14) are the B genome. (JPG 1989 kb) [file 12864_2019_5566_MOESM1_ESM.jpg]
